# Influence of Yoga-Based Personality Development Program on Psychomotor Performance and Self-efficacy in School Children

**DOI:** 10.3389/fped.2016.00062

**Published:** 2016-06-15

**Authors:** Madhusudan Das, Singh Deepeshwar, Pailoor Subramanya, Nandi Krishnamurthy Manjunath

**Affiliations:** ^1^Yoga and Life Sciences Laboratory, Swami Vivekananada Yoga Research Foundation, Bangalore, India

**Keywords:** *yoga*, self-efficacy, attention, trail making test, academic performance

## Abstract

Selective attention and efficacy are important components of scholastic performance in school children. While attempts are being made to introduce new methods to improve academic performance either as part of curricular or extracurricular activities in schools, the success rates are minimal. Hence, this study assessed the effect of *yoga*-based intervention on psychomotor performance and self-efficacy in school children. Two hundred ten school children with ages ranging from 11 to 16 years (mean age ± SD; 13.7 ± 0.8 years) satisfying the inclusion and exclusion criteria were recruited for the 10-day *yogä* program. An equal number of age-matched participants (*n* = 210; mean ± SD; 13.1 ± 0.8 years) were selected for the control group. Participants were assessed for attention and performance at the beginning and end of 10 days using trail making task (TMT) A and B, and self-efficacy questionnaire. The *yoga* group showed higher self-efficacy and improved performance after 10 days of *yoga* intervention. The performance in TMT-A and -B of the *yoga* group showed a significantly higher number of attempts with a reduction in time taken to complete the task and a number of wrong attempts compared with control group. Results suggest that *yoga* practice enhances self-efficacy and processing speed with fine motor coordination, visual–motor integration, visual perception, planning ability, and cognitive performance.

## Introduction

Scholastic performance in school children depends on multiple factors. Memory, attention, and motor speed are some of the important intrinsic factors, which play a major role in an individual’s performance. Motivation to perform can also be influenced by self-efficacy (1). Self-efficacy is defined as “an individual’s judgment of his/her own capabilities to organize and execute the tasks to achieve optimal performance” (2). High self-efficacy is related to a number of positive physical, social, and psychological outcomes. A longitudinal study of 390 adolescents, reported lower levels of depression and delinquency with positively correlated emotional self-efficacy (the perceived ability to handle negative emotions and express positive emotions) (3). Another study on low self-efficacy in students and patients reported severity of social anxiety and associated social impairment (4). Therefore, attempts are being made to create structured activities within the curriculum, which can enhance an individual’s scholastic behavior and performance. But, these programs with focused objectives have resulted in improvement of the student’s performance, but not behavior.

*Yoga* is an ancient Indian discipline that aims at developing an integrated personality, where the growth of physical, mental, social, and spiritual planes is equally focused (5, 6). Manjunath and Telles reported that practicing *yoga* (including postures, regulated breathing, relaxation techniques, and meditation) can improve the ability to plan and execute a given cognitive task in school children (7). Furthermore, several studies have demonstrated the positive impact of *yoga* in terms of enhanced attention, concentration, and memory (visual and spatial memory) (8, 9) in school children. It was demonstrated that the combination of *yoga* practices can improve motor speed (10), while an individual technique (called cyclic meditation) involving focused attention can improve perception (11).

In addition, the beneficial effects of *yoga* in improving hand steadiness, which is suggestive of better attention and concentration (8) has been documented. Furthermore, a recent report suggested an improvement in motor skills and visual discrimination following the practice of a *yoga*-based breathing technique (*Kapalabhati*), compared with breath awareness (12).

It is evident from these earlier studies that combination of *yoga* practices in general, and selected individual *yoga* techniques in isolation, can positively influence an individual’s perception and motor performance. The studies mentioned above provide some evidence for the use of *yoga* in improving motor function, attention, and perception, which are important for scholastic performance. However, there are no comprehensive studies with larger sample size, controlled environment, and longer duration of intervention available to understand the influence of a customized *yoga* module on an individual’s performance and behavior.

Hence, this study was designed to evaluate changes in psychomotor performance and self-efficacy following a specially designed personality development *yoga* camp for school children.

## Materials and Methods

### Participants

Four hundred twenty healthy school children with ages ranging between 13 and 16 years were recruited. Two hundred ten children (M = 13.78; SD = 0.89) were from a self-selected 10-day *yoga*-based “Personality Development Camp” (PDC) at a *yoga* institution, in South India. An equal number of age-matched subjects (M = 13.08; SD = 0.84) without any experience of *yoga* were selected as control group from a school within the same locality. The inclusion criteria included (i) between 13 and 16 years of age, (ii) English as the medium of instruction in the school, (iii) willingness to participate in the study, and (iv) no hospitalization in the last 3 months. The exclusion criteria included (i) any history of neurological or psychiatric disturbances and (ii) learning disability if any (i.e., slow learner). The study was approved by the Institutional Ethics Committee (Res/IEC-SVYASA/003/2013) of the *yoga* institution. Since the selected age group included minors (below 18 years of age), signed informed consent was obtained from their parents and guardian (Principal of the School). Demographic details of the participants are given in Table 1.

**Table 1:** **Characteristics of the participants**.

Participants	Yoga group		Control group	
	*n*	M ± SD	*n*	M ± SD
**Age (years)**	210	13.78 ± 0.89	210	13.08 ± 0.84
**Gender**
Male	140	13.75 ± 0.91	124	13.18 ± 0.83
Female	70	13.84 ± 0.88	86	12.93 ± 0.83
**Socioeconomic status**
High	48 (23%)		32 (15.24%)	
Middle	148 (70.5%)		162 (77.14%)	
Lower	14 (6.5%)		6 (6.7%)	
**Educational status**
Up to 6 years	52 (24.76%)		63 (30%)	
Up to 8 years	123 (58.57%)		119 (56.67%)	
Up to 10 years	35 (16.67%)		28 (13.33%)	

### Design

Eligible subjects from the PDC camp at the *yoga* institution who were willing to participate in the study were stratified based on age and academic status and were assigned to the *yoga* group. The *yoga* group and the control group were assessed before and immediately after 10 days. They were assessed similar to the experimental group on days 1 and 10 during which time the subjects were asked to continue with their normal routine.

### Intervention

The 10-day “Personality Development Camp” (PDC) of 10 h per day was designed for children below 17 years of age. The 10-h routine consisted of physical postures (*Asanas*), voluntary regulated breathing (*Pranayama*), meditation (*Dhyana*), relaxation techniques, internal cleansing practices (*Kriyas*), and reciting hymns from traditional *yoga* texts, music, *yoga* games, and happy assembly. *Kriyas* are yogic cleansing exercises, which are performed to cleanse the body and assist with the natural removal of waste products (13). Details of the intervention are summarized in Table 2.

**Table 2:** **Summarized 10-day *Yoga* intervention program**.

Sl no.	Name of intervention	Duration
1.	*Asana* session Standing posture Sitting postures Prone posture Inverted postures Supine postures	2 h
2.	*Pranayama* session Sectional breathing Thoracic breathing Diaphragm breathing Hyperventilation (*Bhastrika and Kapalbhati*) Balancing of the breath (*NadiShuddhi, AnulomVilom*)	1 h
3.	Cleansing techniques (*Kriyas*) *Trataka* (eye-cleansing techniques) *Sutra Neti* and *JalaNeti* (nasal tract-cleansing techniques) *VamanDhouti* and *LaghuShankaPrakshalana* (GI tract-cleansing techniques)	Twice
4.	*Gita Chanting* session Chanting (18 verses from *Bhagavad Gita*) Yogic discourse	1 h
5.	Creativity hour Karma yoga Designing and arts Tree plantation Debate Stories	2 h
6.	*Bhajan* session Devotional songs Patriotic songs	1 h
7.	Game session Yogic games Group awareness	1 h
8.	Happy assembly Cultural program Team work	2 h

### Assessments

Each subject was assessed in two sessions, one using trail making tasks-A and -B (TMT) (14) and the second using self-efficacy questionnaire for children (SEQ-C) (15). Although trail making tasks are very simple, it has been hypothesized that they reflect various cognitive processes including attention, visual search and scanning, sequencing and shifting, psychomotor speed, abstraction, flexibility, ability to execute and modify a plan of action, and ability to maintain two trains of thoughts simultaneously (12).

During the TMT session, subjects were assessed individually by seating them on a comfortable chair. Trail making task “A” involved subjects drawing lines connecting 25 consecutive circled numbers in a numerical sequence (i.e., 1–2–3, etc.) as rapidly as possible. In task “B,” the subjects were directed to draw lines to connect 12 consecutive circled numbers and 12 consecutive letters in an alternate numeric and alphabetic sequence (i.e., 1–A–2–B, etc.) as rapidly as possible. Time (in seconds) taken for completing the task was noted by using a stop watch. The errors committed during the task completion were scored removing accurately (16).

Self-efficacy questionnaire for children was developed by Muris (15) to assess general self-efficacy across three domains: academic, social, and emotional situations. The SEQ-C is a 24-item self-reported measure with eight items for each domain. Each item is rated on a 5-point Likert scale with 1 being “not at all” and 5 being “very well.” Both assessments were arranged on separate days at the same time for the two groups (i.e., *Yoga* and Control). The SEQ-C was administered as a group test, and the subjects were seated comfortably.

#### Procedure

The TMT and self-efficacy questionnaire (SEQs) questionnaires were administered on children of both the groups (yoga and control) on Day 1 and Day 10 of personality development yoga camp. The data collection lasted for 20 min. The yoga groups underwent for 10 days yoga practices, whereas control group were asked to continue with their normal routine. The yoga educator was blind to the hypothesis of the study. After 10 days of yoga intervention, all children were asked to complete the same questionnaires. The researchers explained about the study and gave stipulated instructions of the manuals to children for better understanding. Children were not given feedback as to their performance on designing experiments or any of the measures.

While data extraction, TMT scores were extracted based on wrong attempts, right attempts, and total attempts to see the error affect of the children. The extraction of TMT also included the completion time for the task, i.e., time duration in seconds. The SEQ-C was extracted based on the responses on three domains of self-efficacy, i.e., (i) social self-efficacy, which has to do with the perceived capability for peer relationship and assertiveness; (ii) academic self-efficacy, which is concerned with the perceived capability to manage one’s own learning behavior, to master academic subjects, and to fulfill academic expectations; and (iii) emotional self-efficacy, which pertains to the perceived capability of coping with negative emotions.

### Data Analysis

The raw data were obtained from Trail Making Tasks A and B as well as SEQ and tabulated separately. The raw data were analyzed using Statistical Package for Social Science (SPSS) Version 20. Data of different variables were tested with the Kolmogorov–Smirnov test for normality. Since, we had two Groups, i.e., *yoga* and control, and two states, i.e., day 1 and day 10, repeated measures analysis of variance (RM-ANOVA) was carried out for each assessment. For Trail making tasks (A and B), RM-ANOVA was performed with one “Within-subjects” factor, i.e., state (day 1 and day 10), which in turn had four subdomains (wrong attempt, right attempt, total attempt, and time taken) and one “Between-subjects” factor, i.e., groups (*yoga* and control). Similarly, for self-efficacy, RM-ANOVA was performed with one “Within-subjects” factor, i.e., states (day 1 and day 10), which in turn had three subdomains (academic, social, and emotional) and one “Between-subjects” factor, i.e., groups (*yoga* and control). This was followed by a pairwise comparison between the mean values of day 1 and day 10 assessments with Bonferroni correction. The alpha level was set at *p* < 0.05.

## Results

### TMT

For Trail making task “A” (TMT-A), the repeated measures ANOVA showed a significant difference for Between-subjects factor, i.e., groups [*F*_(1,418)_ = 12.38; *p* < 0.001; η^2^*p* = 0.029] as well as for Within-subjects factor, i.e., states [*F*_(3,1254)_ = 2164.29; *p* < 0.001; η^2^*p* = 0.84] and for sub domains of TMT-A assessments, i.e., wrong attempt [*F*_(1,418)_ = 29.45; *p* < 0.001; η^2^*p* = 0.06], right attempt [*F*_(1,418)_ = 30.10; *p* < 0.001; η^2^*p* = 0.07], total attempt [*F*_(1,418)_ = 0.55; *p* > 0.05; η^2^*p* = 0.001], and time taken [*F*_(1,418)_ = 58.83; *p* < 0.001; η^2^*p* = 0.13]. The interaction between subdomains of TMT assessments × groups [*F*_(3,1254)_ = 12.24; *p* < 0.001; η^2^*p* = 0.028] and State × Groups [*F*_(1,418)_ = 61.24; *p* < 0.001; η^2^*p* = 0.13] were also significant.

After 10 days, in the Trail making task “A” (TMT-A), pairwise comparisons with Bonferroni correction showed a significant increase in the “right attempt” scores (*p* < 0.001) with the reduction in the “wrong attempt” scores (*p* < 0.001) in the *yoga* group, while there was no difference in the control group. Also, the “total numbers” attempted were significantly higher (*p* < 0.05) and the “time taken” was significantly lower (*p* < 0.001) in the *yoga* group compared with control group.

Similarly, for Trail making task-B (TMT-B), the repeated measures ANOVA showed a significant difference in “Between-subjects” factor, i.e., groups [*F*_(1,418)_ = 12.38; *p* < 0.001; η^2^*p* = 0.029] as well as for “Within-subjects” factor, i.e., states [*F*_(3,1254)_ = 2164.29; *p* < 0.001; η^2^*p* = 0.84], and for sub domain of TMT-B assessments, i.e., wrong attempt [*F*_(1,418)_ = 28.91; *p* < 0.001; η^2^*p* = 0.06], right attempt [*F*_(1,418)_ = 29.7; *p* < 0.001; η^2^*p* = 0.07], total attempt [*F*_(1,418)_ = 1.01; *p* > 0.05; η^2^*p* = 0.002], and time taken [*F*_(1,418)_ = 66.19; *p* = 0.001; η^2^*p* = 0.14]. The interaction between TMT-B assessments × groups [*F*_(3,1254)_ = 12.24; *p* < 0.001; η^2^*p* = 0.028] and state × groups [*F*_(1,418)_ = 61.24; *p* < 0.001; η^2^*p* = 0.13] was also significant.

Pairwise comparisons between mean values of the *yoga* group showed a significant increase in the scores of “right attempts” (*p* < 0.001) with the reduction of “wrong attempts” (*p* < 0.001). Additionally, *yoga* practices, improved “total attempts” (*p* < 0.001) with the reduction in the time taken (*p* < 0.001). On the other hand, there were no significant changes in the control group (*p* > 0.05) scores of day 1 to day 10. The group mean values, SD, median, and interquartile range of trail making task (A and B) of both the groups on day 1 and day 10 are given in Table 3.

**Table 3:** **The groups mean values ±SD of the trail making task (TMT) A and B for yoga and control groups in two states (pre and post)**.

Assessments	Variables	Groups	Pre M ± SD (median)	Post M ± SD (median)	Pre Interquartile range	Post Interquartile range	% Change	T	*p*-Values
**Trail making task A**	Wrong attempt	Yoga	0.61 ± 1.26 (0)	0.05 ± 0.26 (0)	0	0	0.80	0.396	0.000
		Control	0.14 ± 1.88 (0)	0.46 ± 1.01 (0)	0	0	0.65	0.000	0.000
	Right attempt	Yoga	0.39 ± 1.25 (25)	0.95 ± 0.26 (25)	0	0	0.30	−6.379	0.000
		Control	0.87 ± 1.87 (25)	0.54 ± 1.01 (25)	0	0	0.93	−4.934	0.000
	Total attempt	Yoga	0.88 ± 0.76 (25)	0.00 ± 0.07 (25)	0	0	0.48	−2.239	0.026
		Control	0.91 ± 0.60 (25)	0.95 ± 0.44 (25)	0	0	0.17	−0.736	0.463
	Time duration (s)	Yoga	0.92 ± 17.43 (32)	0.48 ± 9.88 (25)	0.5	0	0.28	0.128	0.000
		Control	0.84 ± 26.99 (33)	0.23 ± 22.30 (33)	0	0	0.21	−1.441	0.151
**Trail making task B**	Wrong attempt	Yoga	0.20 ± 2.27 (0)	0.07 ± 0.31 (0)	2	0	0.17	0.329	0.000
		Control	0.51 ± 0.94 (0)	0.36 ± 0.80 (0)	2	0	0.41	0.836	0.068
	Right attempt	Yoga	0.82 ± 2.26 (24)	0.94 ± 0.31 (24)	2	0	0.91	−7.265	0.000
		Control	0.50 ± 0.93 (24)	0.63 ± 0.80 (24)	2	0	0.53	−1.731	0.085
	Total attempt	Yoga	0.73 ± 0.98 (24)	0.98 ± 0.19 (24)	0	0	0.05	−3.722	0.000
		Control	0.93 ± 0.54 (24)	0.00 ± 0.07 (24)	0	0	0.28	−1.672	0.096
	Time duration (s)	Yoga	0.03 ± 28.38 (68)	0.98 ± 15.21 (47)	35.5	19	0.00	0.853	0.000
		Control	0.23 ± 13.38 (58)	0.72 ± 11.87 (60)	30	31	0.17	0.118	0.002

### Self-efficacy

For self-efficacy, the repeated measures ANOVA showed a significant difference “Between-subjects” factor, i.e., groups [*F*_(1,418)_ = 18.94; *p* < 0.001; η^2^*p* = 0.043] and states [*F*_(1,418)_ = 181.29; *p* < 0.001; η^2^*p* = 0.30]. The test of Within-subjects effect showed that there were significant interaction between states × groups [*F*_(1,418)_ = 119.13; *p* < 0.001; η^2^*p* = 0.22] and subdomains of assessments × states [*F*_(3,1254)_ = 107.02; *p* < 0.001; η^2^*p* = 0.20].

The pairwise comparisons with Bonferroni correction for the *yoga* group showed a significant increase in the scores of academic domains (*p* < 0.001), the social domain (*p* < 0.001), and emotional domain (*p* < 0.001), whereas there were no significant changes in the scores of the control group on day 1 to day 10. The group mean values, SD, median, and interquartile range of the SEQ of both the groups on day 1 and day 10 with percentage change (%) are given in Table 4.

**Table 4:** **Descriptive statistics groups mean values ±SD of the self-efficacy questionnaire for yoga and control groups in two states (pre and post)**.

Assessments	Variables	Groups	Pre M ± SD (median)	Post M ± SD (median)	Pre Interquartile range	Post Interquartile range	% Change	*T*	*p*-Values
Self-efficacy questionnaire (SEQ)	Academic domain	Yoga	25.51 ± 7.12 (25)	29.71 ± 5.11 (30)	11	6	15.95	−7.108	0.000
		Control	30.10 ± 6.44 (32)	30.46 ± 6.15 (32)	10	10	1.19	−1.754	0.081
	Social domain	Yoga	24.00 ± 5.94 (23)	28.86 ± 4.63 (29)	8	6	20.25	−10.495	0.000
		Control	27.79 ± 5.48 (28)	28.36 ± 5.25 (29)	8	7	2.05	−2.947	0.004
	Emotional domain	Yoga	22.26 ± 5.65 (21)	27.98 ± 5.00 (28)	7.3	7	25.70	−13.173	0.000
		Control	25.56 ± 5.20 (25)	26.19 ± 4.95 (26)	7.3	7	2.46	−3.318	0.001
	Total score	Yoga	71.96 ± 15.68 (70)	86.66 ± 11.10 (86)	21	13	20.43	−12.725	0.000
		Control	83.49 ± 14.69 (84)	85.04 ± 14.01 (85)	22	21	1.86	−3.812	0.000

The partial correlation showed that there was a significant inverse relationship between total self-efficacy score and the time duration of the test in Trial A (*r* = −0.41; *p* = 0.04) and in Trial B (*r* = −0.21; *p* = 0.003) of *yoga* group, which suggest that with increasing self-efficacy, the duration of the test was reduced in trial A and trial B compared with the control group. This suggests that the self-efficacy was higher in *yoga* group with improved performance as compared with control group.

## Discussion

The results of this study revealed that the school children who performed regular *yoga* practices showed higher self-efficacy and improved performance compared with control group who continued with their normal routine. It also showed a positive relationship between self-efficacy and performance after adjusting for age, gender, and education levels in *yoga* group.

Self-efficacy and executive functions, especially motor performance, play a major role in the scholastic behavior of an individual. Self-efficacy in an individual measures the confidence and ability to execute specific behavior. It suggests that high self-efficacy motivates proactive behavior (17). Hence, this study made an attempt to understand the changes in different dimensions of self-efficacy and executive functions (based on a trail making task).

Our results are consistent with the earlier studies that 10-day *yoga* practice improves visual perception (7) with significant decrease in the number of errors in repeated trials (10, 18). Previous studies demonstrated that the daily *yoga* intervention has a significant impact on key classroom behaviors (19, 20) and improvement in academic performance, especially reading, as well as social and peer interactions in most of the children (21, 22). Berger et al. demonstrated that *yoga* had sustained effects with improvements in behavior, especially in the child’s ability to pay attention in class, organizational skills with homework, and decreased impulsive behavior (23, 24). Recently, a long-term study showed a reduction in time taken to complete neurocognitive tasks after practicing *vihangam yoga* meditation in school children and also suggested that meditation improves mental functions, such as attention span (25), processing speed, attention alternation ability, and performance (26). This may be due to functional reorganization of brain activity patterns for focused attention and cognitive monitoring that takes place with mental practice, and this meditation-related changes are crucially associated to a functional reorganization of activity patterns in prefrontal cortex and in the insula (27). Yogic relaxation and meditation techniques have been shown to improve information processing speed (26, 28) in practitioners. In the context of attention, the TMT have been suggested to tap more complex attention or information processing, immediate memory and performance on recall capacity (29, 30) after *yoga* practice. Telles et al. studied 90 school children (45 in 2 groups) with ages ranging between 9 and 13 years and reported improvement in static motor performance (31) and a significant improvement in motor speed task on 53 adults and 152 children (10) after 10 days yoga intervention. A systematic review article mentioned that *yoga* and meditation intervention nurture mindfulness and may be a feasible and effective method of building resilience in childhood and adolescence (32). Fifty-seven healthy male adult volunteers showed significant improvements in psychomotor task performance (33 34), attention span, processing speed, attention alternation ability, and performance in interference tests (26) after *yoga* practices. *Yoga* may be an effective method to increase awareness and performance in cognitive, emotional, or social behavior (35). Our results are in concurrence with the above studies. All above mentioned studies have a common limitation, i.e., small sample size (21–24), suggesting less statistical power, no proper controlled group, no controlled environment or noise (26), and a short duration of *yoga* practices (1–2 h/day) (21–24). Whereas in this study, we had a large sample size (*n* = 210 in *yoga* and equal participants in controlled group) with controlled environment and comparably long duration of intervention as mentioned above.

The trail making task evaluates fine motor coordination, visual–motor integration, visual perception, and cognitive planning ability (36). Our study reflects the positive effects of *yoga* on academic performance, processing speed, and attention in school children. It was reported earlier that *yoga* practice improves ability of the individual to control visual distraction leading to increased ability for prepotent inhibition or orientational processing (orients to specific objects in the attentional field) (37) and reduces planning time and improves execution time (7). This effect could be a major factor for lowering time duration for matching alphabets with relevant numbers implying that automatic-response generation was lower in the *yoga* group compared with control group in this study. Electroencephalography (EEG) (38) and functional magnetic imaging (39) studies implicated frontal lobe activation during performance of TMT and while matching component of alternating letters of the alphabets and consecutive numbers activated the left dorsolateral prefrontal cortex, precentral gyrus, cingulate gyrus and medial frontal gyrus, and supplementary motor area, which are sensitive to executive functioning (40). These neuroimaging studies suggest that the improvement in the performance of TMT in this study may be due to the improvement of frontal lobe activation in school children following 10 days of intense yoga practice. Another study reported that a short-term yogic practice was associated with a physiological relaxation responses (41), leading to enhanced performance speed on color naming (42), decreased automatic responses (43, 44), and reaction time involving an attentional task (45–48) suggest yoga may enhances parasympathetic dominance with improved frontal lobe activation.

*Yoga* brings about positive changes in behavior and mental health of school children (24, 49). The results of this study are in accordance with the earlier findings indicating a significant relationship of self-efficacy with academic achievements (50). Yoga practices in this study showed positive changes toward the three domains of self-efficacy, i.e., academic domain, social domain, emotional domain, and overall total domain. The social self-efficacy pertains to children’s capability to deal with social challenges, academic self-efficacy refers to children’s perceived capability to master academic affairs, and self-regulatory efficacy deals with children’s capability to resist peer pressure to engage in high risk activities (51). Mindfulness-based mental training study showed reduced level of stress and enhances mindfulness self-efficacy awareness and attention training, and positive states of mind (52). Similarly, this training also testified lowering of psychological distress, such as tension–anxiety, confusion–bewilderment, fatigue–inertia, and vigor–activity parameters in medical students (53). Further studies on yoga practices showed improvement in greater awareness of the feelings associated with stress, and it may enhance coping abilities possibly as part of the process of developing mindfulness or related to cognitive, emotional, or social development (23, 54, 55). This leads to academic performance, alertness, and academic excellence that are concerned with the quantity and quality of learning attained with yoga or mental training intervention. The academic performance and alertness in school children was high who practiced yoga and reported low stress in children (56). The regular practice of yoga can improve Sattva Guna (balanced personality trait) among students paving the way for their academic excellence. The academic excellence is essential to provide opportunities for students to work together to improve their understanding of concepts in their academic core that helps students to train problem solving and collaborative learning strategies (6). Another major component for higher academic performance is better planning ability with self-regulatory behavior (57). Studies suggest that yoga-based education program improve planning and execution skills in school boys (58). In addition to this, the beneficial effects of meditation on middle school children showed an increased state of restful alertness and greater capacity for self-reflection, self-control, and flexibility as well as improved academic performance that may facilitate the growth of social–emotional capacities necessary for regulating the emotional labiality and interpersonal stress of adolescence (59).

The findings of this study extended previous research on either self-efficacy or psychomotor performance in children, which were assessed simultaneously in this study. Yoga group children reported higher self-efficacy particularly in academic, social, and emotional domain with greater improvement in performance of psychomotor skills and executive functions, which require selective attention, concentration, and visual scanning abilities, and reduced the planning and execution time in TMT task.

**Figure 1:**
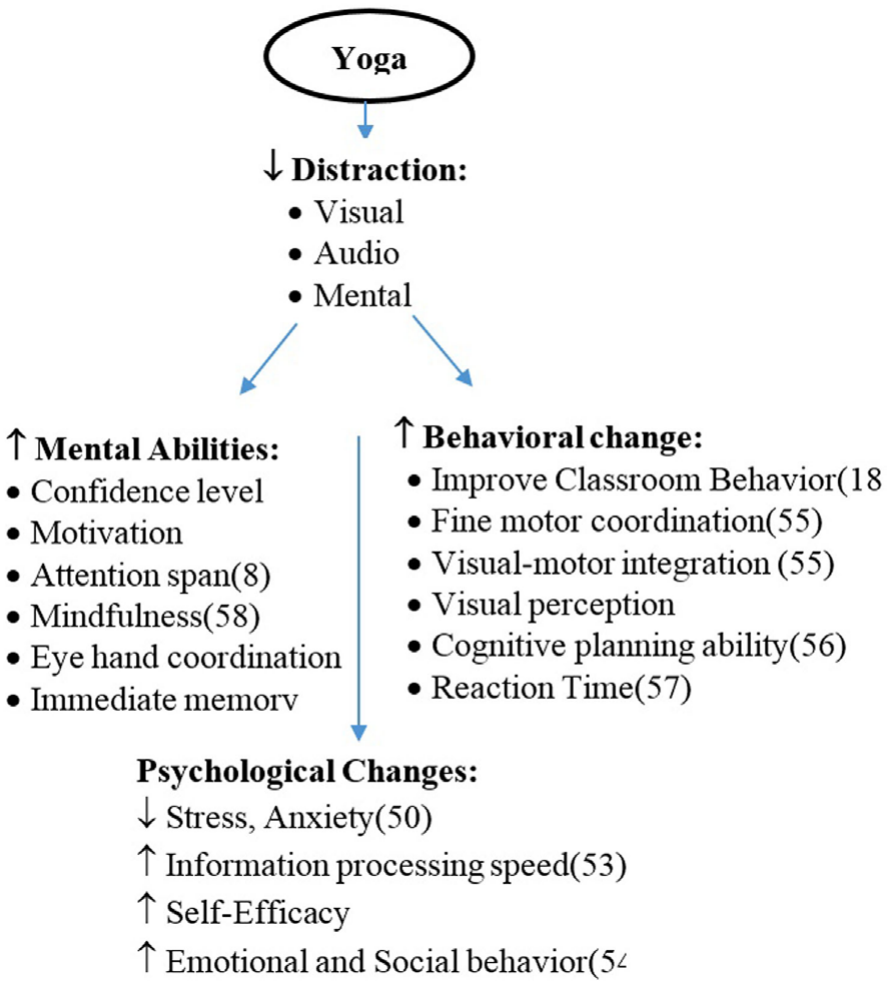
**Evidence-based beneficial effects of yoga in school children**.

Evidence-based previous scientific studies supporting the findings of the preset study on the effect of yogic practices on psychological, behavioral, and cognitive abilities in children are given in Figure 1. Yoga practices reduce visual, audio, and mental distractions, which helps in reducing stress and anxiety in children. Once mental distraction reduced, the mental abilities will be increased with the improvement in their behavioral skills.

The findings of this study are interesting and straightforward, and the study has a major limitation in the selection of school children that was not random in the control group because of unavailability of non-*yoga* practitioner children in the *yoga* institute. There was also no follow-up, and so, it is not known whether improvements in test performance and self-efficacy are sustained. Despite of aforementioned limitations, the results of our study suggest that *yoga*-based intervention in school children can improve attention, motor function, and different domains of personality. Initial research on the usefulness of *yoga* for children and adolescents is promising, more systematic studies including long-term randomized controlled trials (RCTs) with follow-up and active control groups are needed.

## Author Contributions

All authors listed have made substantial, direct, and intellectual contribution to the work and approved it for publication.

## Conflict of Interest Statement

The authors declare that the research was conducted in the absence of any commercial or financial relationships that could be construed as a potential conflict of interest.
