# Supplementary material for: Influence of Yoga-Based Personality Development Program on Psychomotor Performance and Self-efficacy in School Children
Source: Front Pediatr. 2016 Jun 15;4:62. doi: 10.3389/fped.2016.00062 (PMC4908105; doi:10.3389/fped.2016.00062)

Trail Making Test Part A

Patient's Name: ________________________________ Date: ______________________

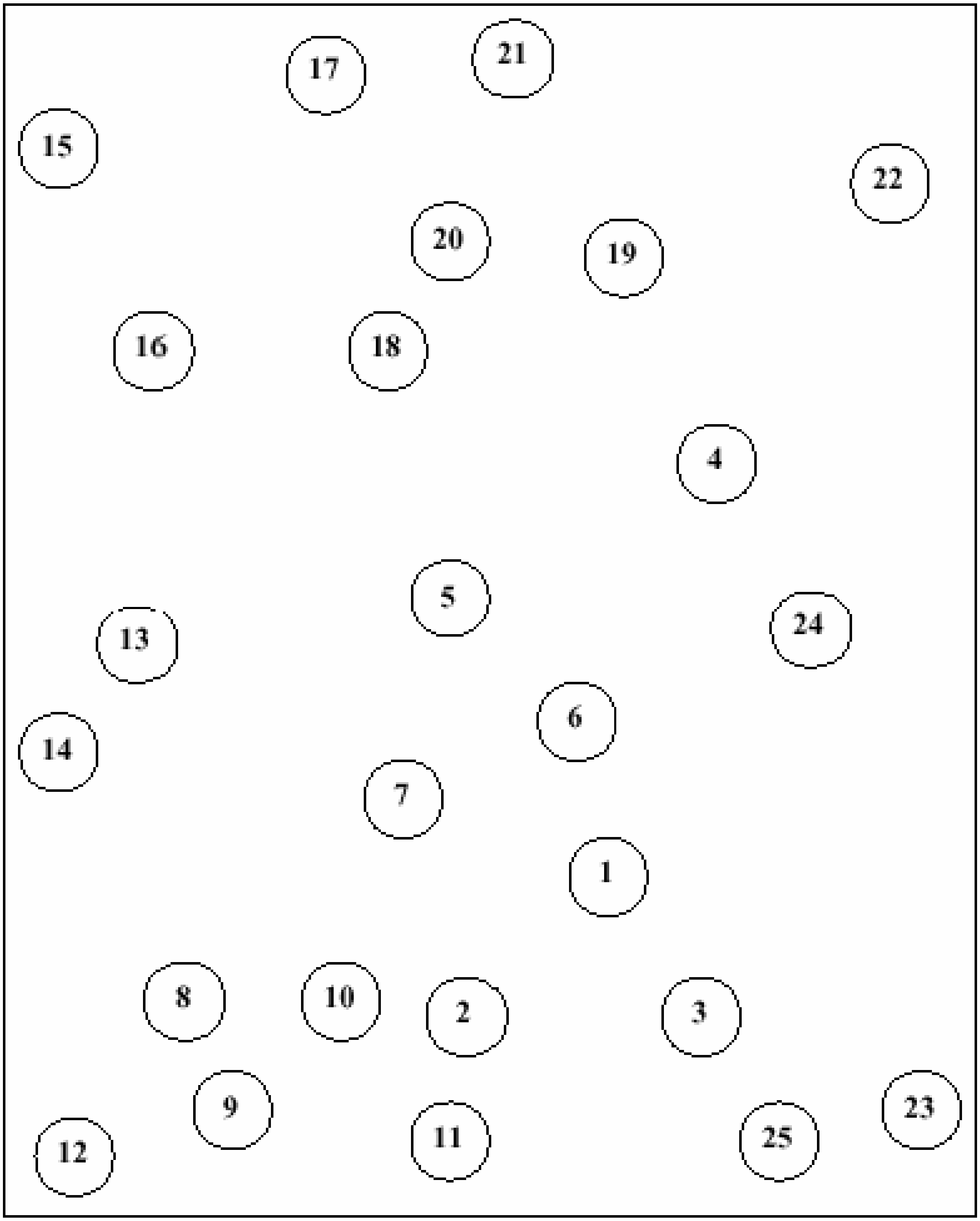

Trail Making Test Part A – *SAMPLE*

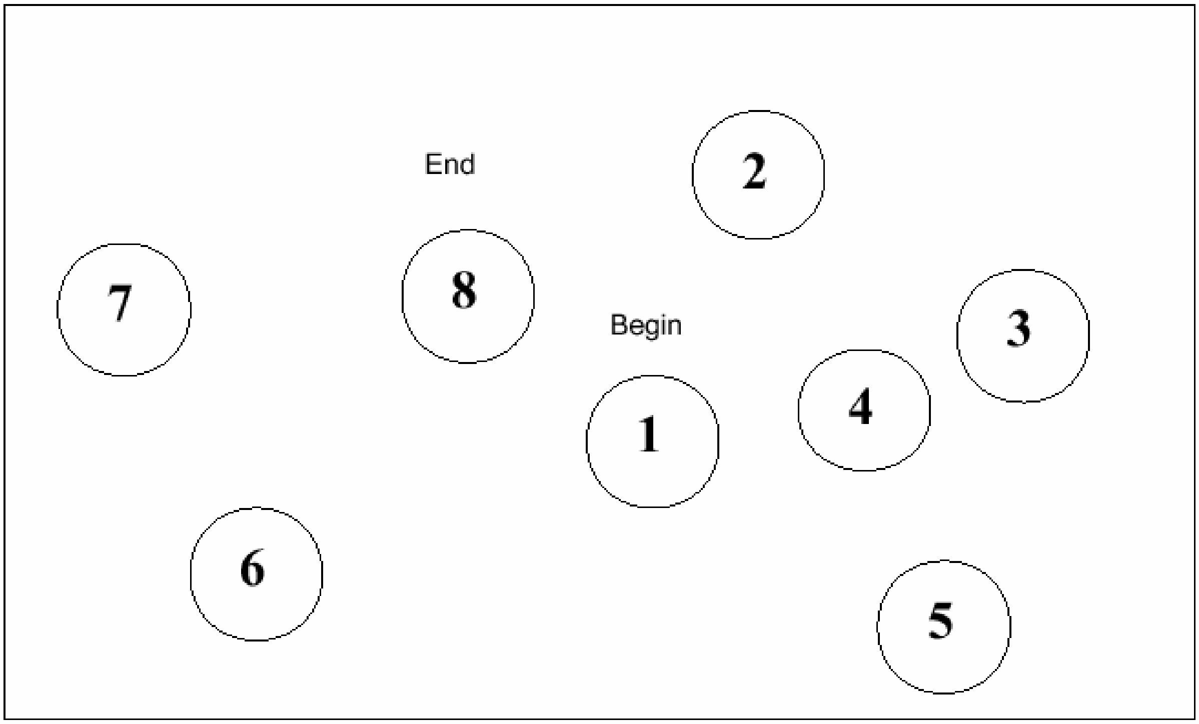

Trail Making Test Part B

Patient's Name: ________________________________ Date: ______________________

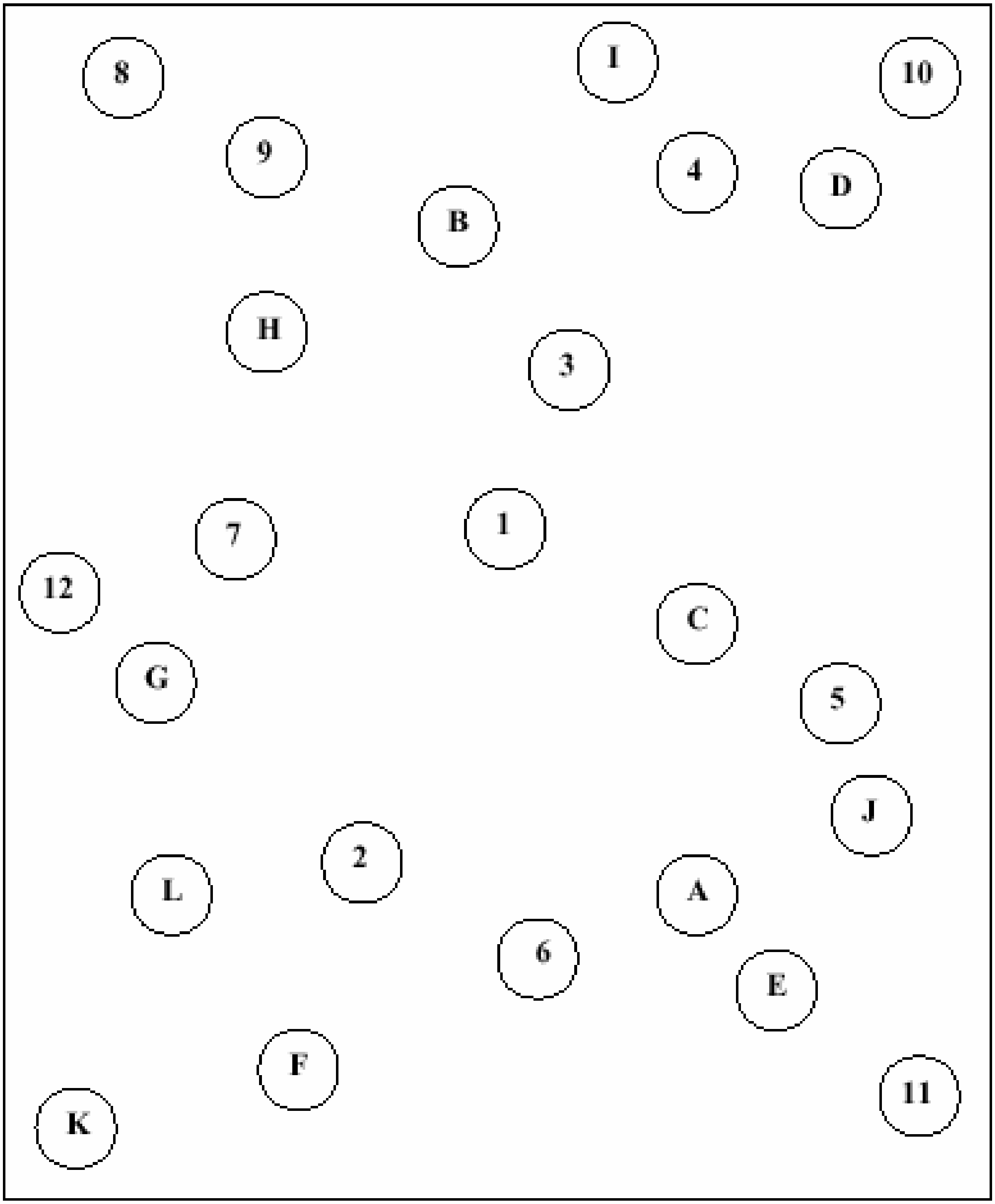

Trail Making Test Part B – *SAMPLE*

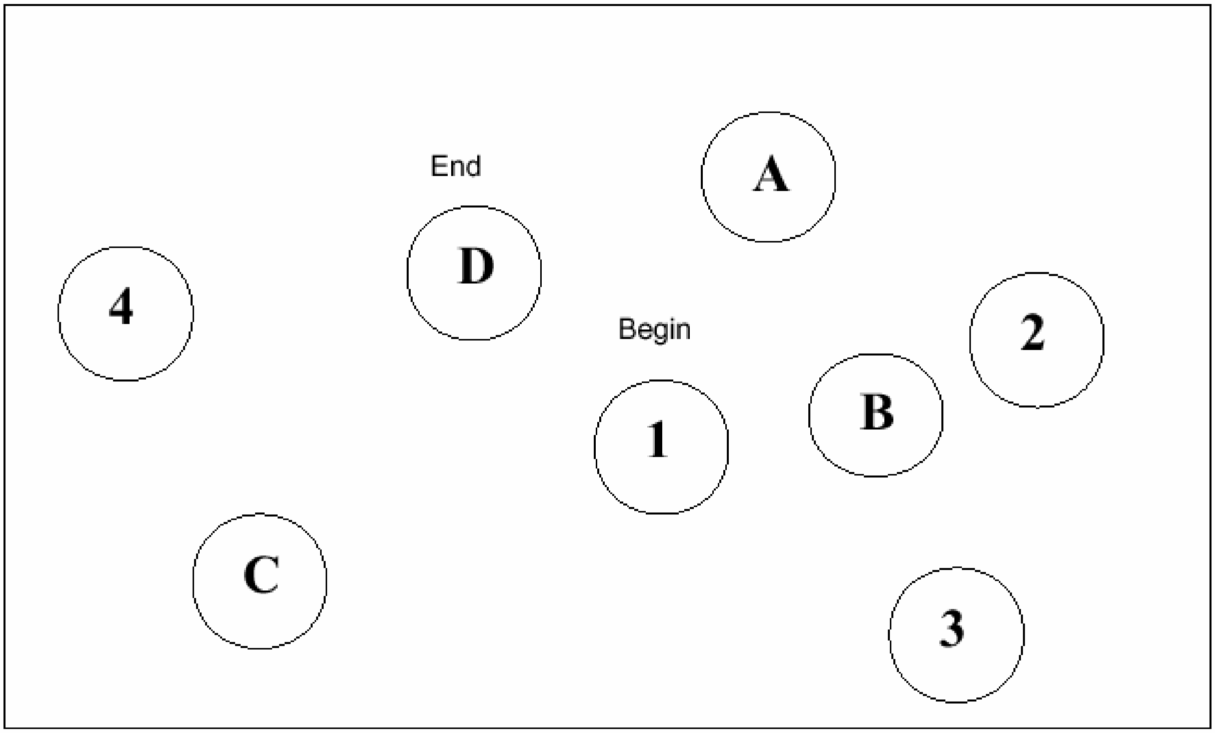

Supplement: Supplementary file 1 [file image_1.pdf]
