# Supplementary material for: Influence of Yoga-Based Personality Development Program on Psychomotor Performance and Self-efficacy in School Children
Source: Front Pediatr. 2016 Jun 15;4:62. doi: 10.3389/fped.2016.00062 (PMC4908105; doi:10.3389/fped.2016.00062)
Supplement: Supplementary file 2 [file image_2.pdf]

Self-Efficacy Questionnaire for Children (SEQ-C)
Brief Survey on Academic, Social and Emotional Self-Efficacy

		Not at all 1	2	3	4	Very well 5
1.	How well can you get teachers to help you when you get stuck on schoolwork?	O	O	O	O	O
2.	How well can you express your opinions when other classmates disagree with you?	O	O	O	O	O
3.	How well do you succeed in cheering yourself up when an unpleasant event has happened?	O	O	O	O	O
4.	How well can you study when there are other interesting things to do?	O	O	O	O	O
5.	How well do you succeed in becoming calm again when you are very scared?	O	O	O	O	O
6.	How well can you become friends with other children?	O	O	O	O	O
7.	How well can you study a chapter for a test?	O	O	O	O	O
8.	How well can you have a chat with an unfamiliar person?	O	O	O	O	O
9.	How well can you prevent to become nervous?	O	O	O	O	O
10.	How well do you succeed in finishing all your homework every day?	O	O	O	O	O
11.	How well can you work in harmony with your classmates?	O	O	O	O	O
12.	How well can you control your feelings?	O	O	O	O	O
13.	How well can you pay attention during every class?	O	O	O	O	O
14.	How well can you tell other children that they are doing something that you don’t like?	O	O	O	O	O
15.	How well can you give yourself a pep-talk when you feel low?	O	O	O	O	O
16.	How well do you succeed in understanding all subjects in school?	O	O	O	O	O
17.	How well can you tell a funny event to a group of children?	O	O	O	O	O
18.	How well can you tell a friend that you don’t feel well?	O	O	O	O	O
19.	How well do you succeed in satisfying your parents with your schoolwork?	O	O	O	O	O
20.	How well do you succeed in staying friends with other children?	O	O	O	O	O
21.	How well do you succeed in suppressing					

	unpleasant thoughts?	○	○	○	○	○
22.	How well do you succeed in passing a test?	○	○	○	○	○
23.	How well do you succeed in preventing quarrels with other children?	○	○	○	○	○
24.	How well do you succeed in not worrying about things that might happen?	○	○	○	○	○

Scoring

A total self-efficacy score can be obtained by summing across all items.
Items 1, 4, 7, 10, 13, 16, 19, and 22 = Academic self-efficacy
Items 2, 6, 8, 11, 14, 17, 20, and 23 = Social self-efficacy
Items 3, 5, 9, 12, 15, 18, 21, and 24 = Emotional self-efficacy
